# Evaluating the Accessibility of Healthcare Facilities Using an Integrated Catchment Area Approach

**DOI:** 10.3390/ijerph15092051

**Published:** 2018-09-19

**Authors:** Xiaofang Pan, Mei-Po Kwan, Lin Yang, Shunping Zhou, Zejun Zuo, Bo Wan

**Affiliations:** 1 Faculty of Information Engineering, China University of Geosciences, 388 Lumo Road, Wuhan 430074, China; xfpanem@163.com (X.P.); zhoushunping@mapgis.com (S.Z.); zuozejun@mapgis.com (Z.Z.); magicwan1105@163.com (B.W.); 2 School of Geographic Sciences, Xinyang Normal University, 237 Nanhu Road, Xinyang 464000, China; 3 Department of Geography and Geographic Information Science, University of Illinois at Urbana-Champaign, Natural History Building, MC-150, 1301 W Green Street, Urbana, IL 61801, USA; mpk654@gmail.com; 4 Department of Human Geography and Spatial Planning, Utrecht University, P.O. Box 80125, 3508 TC Utrecht, The Netherlands; 5 State Key Laboratory of Geo-information Engineering, Xi’an 710054, China

**Keywords:** healthcare accessibility, catchment areas, access probability, taxi GPS trajectories, E2SFCA

## Abstract

Accessibility is a major method for evaluating the distribution of service facilities and identifying areas in shortage of service. Traditional accessibility methods, however, are largely model-based and do not consider the actual utilization of services, which may lead to results that are different from those obtained when people’s actual behaviors are taken into account. Based on taxi GPS trajectory data, this paper proposed a novel integrated catchment area (ICA) that integrates actual human travel behavior to evaluate the accessibility to healthcare facilities in Shenzhen, China, using the enhanced two-step floating catchment area (E2SFCA) method. This method is called the E2SFCA-ICA method. First, access probability is proposed to depict the probability of visiting a healthcare facility. Then, integrated access probability (IAP), which integrates model-based access probability (MAP) and data-based access probability (DAP), is presented. Under the constraint of IAP, ICA is generated and divided into distinct subzones. Finally, the ICA and subzones are incorporated into the E2SFCA method to evaluate the accessibility of the top-tier hospitals in Shenzhen, China. The results show that the ICA not only reduces the differences between model-based catchment areas and data-based catchment areas, but also distinguishes the core catchment area, stable catchment area, uncertain catchment area and remote catchment area of healthcare facilities. The study also found that the accessibility of Shenzhen’s top-tier hospitals obtained with traditional catchment areas tends to be overestimated and more unequally distributed in space when compared to the accessibility obtained with integrated catchment areas.

## 1. Introduction

Accessibility refers to the ease with which activity locations or urban services can be reached from a particular location or by an individual [1,2,3,4,5]. Location-based or place-based accessibility—which is conceptually different from individual-based accessibility—is widely used in assessing whether the spatial distribution of public facilities is optimal or not [6]. As an important component of public facilities, healthcare facilities provide residents with necessary medical services, and their distribution and accessibility affect residents’ well-being directly. Good accessibility to healthcare facilities for every person is an essential goal for governments [7,8]. Thus, healthcare service planners need more accurate and reliable methods for finding deficiencies in the accessibility of health services [9,10].

Besides distance-based models, physician-to-population ratio-based models and gravity models, the two-step floating catchment area (2SFCA) method proposed by Luo and Wang [4] has grown in prominence in the last decade for evaluating the accessibility of healthcare [8]. In the 2SFCA method, a floating catchment area is used as a “window” in which residents may visit physicians, who in turn can serve those residents within the “window”. Therefore, a physician-to-population ratio can be calculated for each health service, and then all the physician-to-population ratios can be summed up for each location within the catchment area and used as the accessibility of that location.

However, the 2SFCA method has two implicit limitations. One is that it uses dichotomies to deal with people’s demands and another is that it does not consider distance decay within a catchment area [10]. In other words, people within a catchment area can access the physicians within it while people outside the catchment area cannot; besides, people within a catchment area have equal access to the physicians [10,11,12,13]. In real-world situations, distance from the health facilities within a catchment area may affect people’s choice and behavior, and there are no hard boundaries that prevent people from using healthcare facilities in catchment areas outside of their own ones. To address these shortcomings, a distance-decay function has been used in recent studies as an additional component of the 2SFCA method [10,13,14] which includes, for example, the kernel density function [15] and the Gaussian function [16]. Luo and Qi [10] suggested that people would not mind a few minutes of difference in travel time to seek medical care, and proposed an enhanced two-step floating catchment area (E2SFCA) method which uses multiple distance decay weights that substitute the dichotomous scheme (zero and one) of the 2SFCA method. As an improvement of the 2SFCA method, a catchment area in the E2SFCA method is divided into several subzones according to the spatial barrier between residents and health services. For instance, a 30-min catchment area is divided into three subzones with breaks at 10 and 20 min [10,11], or a 180-min catchment area is divided into four subzones with breaks at 30, 60, and 120 min [12]. After that, the multiple distance decay weights are assigned to different subzones for calculating the physician-to-population ratio and accessibility.

Thus, in the enhanced two-step floating catchment area (E2SFCA) method, the catchments and subzones of healthcare facilities are artificially set according to the spatial barriers in different cases. To be consistent with the actual traffic conditions of different applications, travel time or travel distance based on the road network is often used as the spatial barrier or impedance [17]. Then accessibility is calculated based on the catchment areas generated based on the spatial barrier or impedance. However, in the real world, people who benefit from health services are affected by various factors in addition to spatial barriers, such as the effectiveness of services, attractiveness of the facility, and sociocultural or socioeconomic barriers [18,19,20]. For example, Dony et al. [20] proposed that the catchment area of a service facility is a function of its attractiveness. In a study that compares the potential and revealed access to healthcare facilities, Casas et al. [21] found that real travel time to the closest hospital is nearly six times longer than estimated travel time during the dengue fever outbreaks, which suggests that the healthcare facility where healthcare is received rarely coincides with the closest one. The study indicates that patients often prefer to visit healthcare facilities farther away from home instead of closer ones, and this is a common phenomenon. This bypass behavior [22] results in some users of the healthcare facilities in a catchment area not being included in it or some residents of a particular catchment area not visiting the facilities within it. In other words, the arbitrarily defined catchments and subzones, which are based on theoretical spatial barriers, may not represent the actual distribution of patients for the healthcare facilities in a study area. As Wang [23] suggested, any debate over the right size of catchment areas cannot be settled without analyzing real-world healthcare utilization patterns. Therefore, the catchments and subzones area in the E2SFCA method should be adjusted according to real healthcare access behavior.

Questionnaire data or patient information obtained from hospitals are often used as the data source for identifying actual catchment areas [24,25,26,27]. Nevertheless, large amounts of data about real patients are hard to obtain in many countries, including China, because of privacy concerns and commercial interests. In recent years, GPS trajectory data on the movement of taxis are increasingly used in social science and transportation studies [28,29,30,31,32]. As a common travel mode, especially in metropolitan areas, taxi travel accounts for a considerable proportion of total travel in China. For instance, from 2010 to 2016, the shares of taxi travel in total transport in Beijing, Shanghai, Guangzhou and Shenzhen (the top four metropolitan areas in China) were 8%, 16.5%, 13.8% and 12.2% respectively, according to statistical yearbooks data [33,34,35,36]. Similar to private cars, taxis are more convenient than other public transportation modes, so taxi becomes the first choice for people with medical emergencies or visiting hospitals when private cars are not available [37]. According to a report from the Didi Media Research Institute and CBNData (the biggest online taxi transaction platform in China), about 9.23% of taxi travel involves hospital visits [38]. This indicates that about 10% of taxi GPS tracks can be used to represent riders’ actual visits to urban medical facilities.

To address the limitations of previous E2SFCA methods, this study uses taxi GPS trajectories to incorporate the access relationships between actual users and healthcare facilities in the assessment of the accessibility of these facilities. The catchment areas of medical facilities are adjusted by integrating actual access behaviors with theoretical catchments, and these adjusted catchment areas are then incorporated into the E2SFCA method to evaluate the accessibility of healthcare facilities. In comparison with previous studies, this study seeks to contribute to the literature in three main ways: (1) it proposes using access probability to depict people’s access behaviors to healthcare facilities from the theoretical, actual, and integrated perspectives; (2) it formulates the notion of integrated catchment areas (ICA) to approximate the catchment areas of healthcare facilities through the constraint of integrated access probability instead of traditional spatial barriers; and (3) by introducing the ICA into the E2SFCA method and incorporating an actual access element in catchment area delineation, the resulting accessibility levels are more in line with people’s actual behavior.

## 2. Method

### 2.1. Conceptual Framework

**Figure 1:**
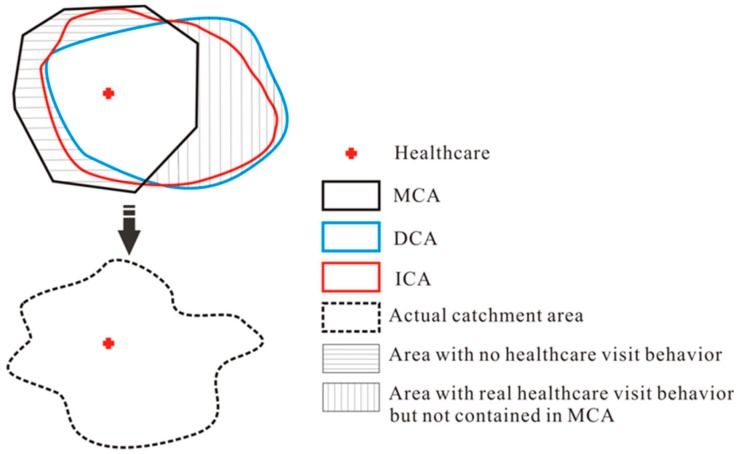
The relationship among model-based catchment area (MCA), data-based catchment area (DCA), integrated catchment area (ICA) and actual catchment area.

This study seeks to develop a method for delineating the catchment areas of healthcare facilities that takes into account people’s actual hospital visit behaviors. As discussed earlier, a theoretical catchment area based on distance or travel time may not include some areas in which patients within the catchment area patronize physicians, or may contain some locations where no healthcare visits occur. Meanwhile, pertinent taxi trajectories can be used to identify the actual origins and destinations of real patients and thus address the limitations of traditional catchments to a certain extent. Theoretical (or model-based) catchments and trajectory data-based catchments are deployed in this study as two complementary perspectives. They are integrated to generate catchment areas that better reflect people’s actual visits to healthcare facilities. The relationships between them are shown in Figure 1. A model-based catchment area (MCA) delineates the catchment area of a medical facility using theoretical distance. A data-based catchment area (DCA) is derived from a big taxi trajectory dataset. The MCA and DCA can be integrated to obtain the integrated catchment area (ICA), which adjusts the MCA to take into account actual hospital visit behavior. Through such integration, the ICA better reflects people’s behavior and is used as the “window” in the E2SFCA method in this study.

In previous studies, if a location is within a spatial barrier threshold, it is regarded as inside the catchment area, and if a location is beyond this threshold, it is regarded as outside the catchment area. However, some residents within a catchment area may not utilize the healthcare facilities within it while some people living outside this catchment area may utilize these facilities. Therefore, in this paper, whether a location belongs to a catchment area or not depends on the probability of people who live at that location visiting the facilities within the catchment. In light of the flexibility of people’s healthcare choices, every healthcare facility has a possibility of being visited by any patient. We thus define this possibility of visit as access probability. Access probability is in the range of zero to one and the value shows the likelihood of visiting a healthcare facility by people at a specified location. Note that access probability generated from conventional models and actual data are not the same, which are defined as MAP (model-based access probability) and DAP (data-based access probability) respectively, while integrated access probability (IAP) refers to the likelihood of visiting a healthcare facility considering both model-based and data-based perspectives. In what follows, different access probability indexes are defined in detail.

### 2.2. The Access Probability Indexes

Model-based access probability (MAP) is the access probability based on a spatial distance threshold. Ingram proposed an accessibility model and suggested that the shorter the distance from a specific location to a service facility, the higher the access probability of the location [39]. Furthermore, distance decay is an important notion in geography [40,41]. Therefore, according to the principles of least effort and distance decay, people tend to choose the nearest facility, and the probability of visiting a facility decreases as distance increases [27,42,43]. This means that the shorter the distance, the higher the MAP. Therefore, an inverted power function which is similar to a distance decay function is used in the MAP.

Suppose *SA* is the study area, let *F* = {*f*_1_, *f*_2_, …, *f_n_*} be the set of healthcare facilities in *SA*, and *k* be any location within *SA*. The MAP of the resident at location *k* visiting a healthcare facility *f_i_* can be expressed as Formula (1):

(1) $$MAP(\alpha_{k}^{f_{i}})=\frac{1/d_{i}}{\sum_{i=1}^{n}1/d_{i}}$$

and Formula (1) satisfies the constraint of Formula (2):

(2) $$MAP(\alpha_{k}^{f_{1}})+MAP(\alpha_{k}^{f_{2}})+\ldots +MAP(\alpha_{k}^{f_{n}})=1,$$

**Figure 2:**
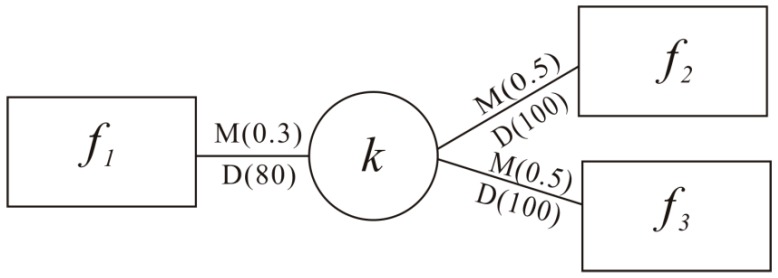
A scenario of model-based and data-based visiting probability to healthcare facilities.

In Formula (1), *d_i_* denotes the distance from *k* to *f_i_* and *n* denotes the total number of facilities in the study area. For instance, as Figure 2 shows, the travel distance from location *k* to facilities *f*_1_, *f*_2_, *f*_3_ are represented by the inverted power function weight (in parentheses on the line). The MAP of location *k* to the three healthcare facilities are: $MAP\left(\alpha_{k}^{f_{1}}\right)=\frac{0.3}{0.3+0.5+0.5}=0.231$, $MAP\left(\alpha_{k}^{f_{2}}\right)=\frac{0.5}{0.3+0.5+0.5}=0.385$, and $MAP\left(\alpha_{k}^{f_{3}}\right)=\frac{0.5}{0.3+0.5+0.5}=0.385$.

However, data-based access probability (DAP), which denotes the actual visit probability based on taxi trajectory data, differs. It is defined by the number of passengers who get off at a healthcare facility. The more passengers that get off at a facility, the greater attractiveness and the higher actual access probability it has [6,44]. Therefore, the DAP of one location to a particular healthcare facility can be denoted as the percentage of patients who actually visited this facility among all patients that originated from that location. DAP is thus represented by Formula (3):

(3) $$DAP(\alpha_{k}^{f_{i}})=\frac{p_{i}}{\sum_{i=1}^{n}p_{i}}$$

Similarly, $DAP(\alpha_{k}^{f_{i}})$ satisfies Formula (4):

(4) $$DAP(\alpha_{k}^{f_{1}})+DAP(\alpha_{k}^{f_{2}})+\ldots +DAP(\alpha_{k}^{f_{n}})=1$$

In Formula (3), *p_i_* denotes the number of residents who visit *f_i_* from *k* and *n* denotes the total facilities number in the study area. As shown in Figure 2, the number of residents from *k* to *f*_1_, *f*_2_, *f*_3_ are shown in parentheses below the line. Therefore, $DAP\left(\alpha_{k}^{f_{1}}\right)=\frac{80}{80+100+100}=0.286$, $DAP\left(\alpha_{k}^{f_{2}}\right)=\frac{100}{80+100+100}=0.357$, and $DAP\left(\alpha_{k}^{f_{3}}\right)=\frac{100}{80+100+100}=0.357$.

MAP and DAP describe the access probability to a medical facility from two different perspectives. According to Section 2.1 above, integrated access probability (IAP) is defined as the combination of MAP and DAP. Obviously, an increase in both MAP and DAP leads to an increase in IAP (i.e., IAP is positively correlated with MAP and DAP). To ensure that the value of IAP is in the range of zero to one, IAP is formulated as a linear function of DAP and MAP with different weights. Therefore, the IAP of location *k* to healthcare *f_i_* is expressed as Formula (5):

(5) $$IAP\left(\alpha_{k}^{f_{i}}\right)=\lambda_{1}MAP\left(\alpha_{k}^{f_{i}}\right)+\lambda_{2}DAP\left(\alpha_{k}^{f_{i}}\right)$$

In Formula (5), *λ*_1_, *λ*_2_ denotes the weight of MAP and DAP respectively. If there are no residents visiting *f_i_*, *p_i_* is equal to zero and thus $DAP(\alpha_{k}^{f_{i}})$ is equal to zero. In this case, $IAP(\alpha_{k}^{f_{i}})$ is determined only by $MAP(\alpha_{k}^{f_{i}})$, which is the same as the traditional methods.

### 2.3. The Integrated Catchment Area

The criterion for determining whether a location falls within the catchment area of a medical facility is the access probability of this location being greater than or equal to a certain threshold [45]. All the locations that satisfy the condition constitute the catchment area of this facility.

Assume *δ* is the access probability threshold, the catchment of the facility *f_i_* under *δ* is the collection of location *k* whose access probability is greater than or equal to *δ*. Therefore, three catchment areas, MCA, DCA and ICA are formed as Formulas (6)–(8):

(6) $$MCA\left(f_{i},\delta \right)=\{k|MAP\left(\alpha_{k}^{f_{i}}\right)\geq \delta ,k\in SA,f_{i}\in F,\delta \in \left[0,1\right]\},$$

(7) $$DCA\left(f_{i},\delta \right)=\left\{k|DAP\left(\alpha_{k}^{f_{i}}\right)\geq \delta ,k\in SA,f_{i}\in F,\delta \in \left[0,1\right]\right\},$$

(8) $$ICA\left(f_{i},\delta \right)=\left\{k|IAP\left(\alpha_{k}^{f_{i}}\right)\geq \delta ,k\in SA,f_{i}\in F,\delta \in \left[0,1\right]\right\},$$

In Formulas (6)–(8), *δ* is the access probability threshold in the range of zero to one. $MCA\left(f_{i},\delta \right)$, $DCA\left(f_{i},\delta \right)$ and $ICA\left(f_{i},\delta \right)$ denote the model-based, data-based, and integrated catchment areas of a healthcare facility that satisfy the constraint of *δ*. Due to the difference in access probability defined in Formulas (6)–(8), these three catchment areas are different at the same threshold. Moreover, different thresholds generate different results for each kind of catchment area, which means the catchment area varies with a change in the access probability threshold. In other words, if a set of continuous thresholds are given, a collection of catchment areas representing different access probabilities will be generated.

### 2.4. Accessibility Measurement Based on ICA

In order to differentiate the accessibility within a catchment area, multiple travel time zones within each catchment area are obtained and assigned with different weights in the E2SFCA method [10]. In this study, the integrated catchment area (ICA) is used in the E2SFCA method. Therefore, we name this method the E2SFCA-ICA method. In this method, the subzones are identified by different IAP thresholds. Given that the study area is covered by a grid structure and each grid represents a population unit, the E2SFCA-ICA method is implemented in two steps:

Step1: $ICA\left(f_{i},\delta \right)$ is defined as the catchment area of facility *f_i_* with IAP of *δ*. Then, each catchment area is divided into multiple subzones of *subzone*_1_, *subzone*_2_, …, *subzone_r_* when the thresholds of IAP are *δ*_1_, *δ*_2_, …, *δ_r_*, respectively. Search all population units (here, a grid cell) that are within *subzone_j_* from facility *f_i_* and compute the weighted physician-to-population ratio *R_i_*, which is represented by Formula (9):

(9) $$R_{i}=\frac{S_{i}}{\sum_{k\in ICA\left(f_{i},\delta_{j}\right)}pop_{k}w_{j}}\;=\frac{S_{i}}{\sum_{k\in ICA\left(f_{i},\delta_{1}\right)}pop_{k}w_{1}+\sum_{k\in ICA\left(f_{i},\delta_{2}\right)}pop_{k}w_{2}+\ldots +\sum_{k\in ICA\left(f_{i},\delta_{r}\right)}pop_{k}w_{r}}$$

where *δ_j_* is the IAP threshold of *subzone_j_*, $ICA\left(f_{i},\delta_{j}\right)$ is the integrated catchment area (ICA) of *f_i_* under *δ_j_*, *pop_k_* denotes the population unit of location *k* falling within $ICA\left(f_{i},\delta_{j}\right)$ and *S_i_* is the number of physicians in a healthcare facility *f_i_*, and *w_r_* is the distance weight for *r_th_* subzone.

Step 2: For each location *k*, search all healthcare facilities that are within $ICA\left(f_{i},\delta_{j}\right)$ and sum up all of the physician-to-population ratios as its accessibility, which is represented by Formula (10):

(10) $$A_{k}=\sum_{i\in ICA\left(f_{i},\delta_{j}\right)}R_{i}w_{j}\;=\sum_{i\in ICA\left(f_{i},\delta_{1}\right)}R_{i}w_{1}+\sum_{i\in ICA\left(f_{i},\delta_{2}\right)}R_{i}w_{2}+\ldots +\sum_{i\in ICA\left(f_{i},\delta_{r}\right)}R_{i}w_{r},$$

where *A_k_* represents the accessibility of location *k*, and *R_i_* denotes the physician-to-population ratio of facility *f_i_* that falls within the catchment area centered at population *k*. The same IAP threshold of subzone and distance weights in Step 1 are applied in Step 2.

The greater the value of *A_k_*, the higher the accessibility of location *k* is to healthcare facilities. The smaller the differences in accessibility between different locations, the more equitable the distribution of healthcare facilities is, and vice versa. The advantage of this method is that the catchment areas and subzones are determined by the characteristics of the specific study area instead of an arbitrary value.

## 3. Case Study

### 3.1. Study Area

The study area for this research is Shenzhen, a metropolitan area in southern Guangdong Province in China. Shenzhen is comprised of six districts (Futian, Luohu, Nanshan, Yantian, Baoan, and Longgang) with a total area of 1996.85 km^2^ and a permanent population of about 10.35 million in 2010 according to the Sixth National Census of China. The population of each sub-district is obtained from the Shenzhen statistical yearbook (http://www.sztj.gov.cn/). The year of the road network used in this study is 2010.

**Figure 3:**
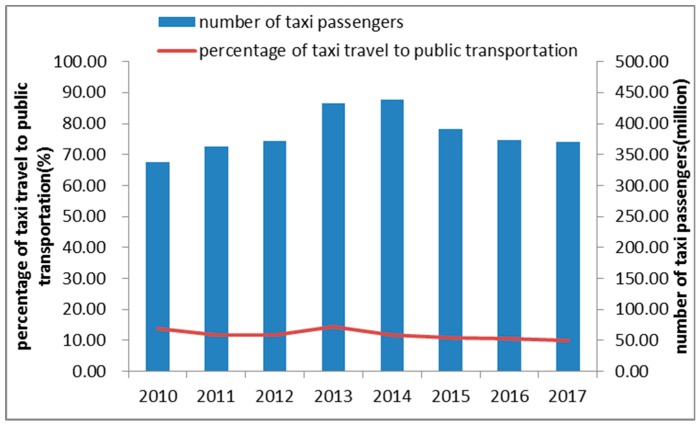
Taxi travel statistics of Shenzhen from 2010 to 2017.

Taxis play an important role in public transportation in Shenzhen. According to a report from the Shenzhen Transportation Commission (http://www.sztb.gov.cn/), the number of taxi passengers exceeded 350 million each year from 2010 to 2017, which accounted for an average of 12.2% of travel by public transportation annually (Figure 3). In this study, the taxi trajectory dataset was collected from 12,448 taxis from 1 November to 7 November 2011. The weather in Shenzhen is comfortable in November, so passengers do not take taxis to protect themselves from storms or cold weather. Meanwhile, there are no holidays on these days, so taxis operated normally in this period.

**Figure 4:**
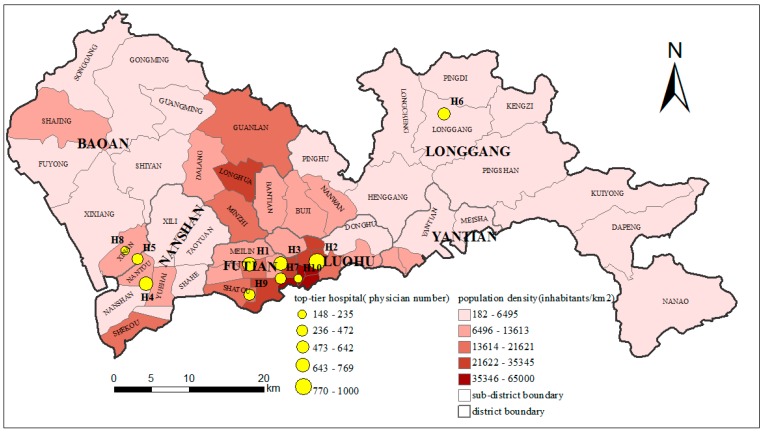
The top-tier hospitals and the population density of Shenzhen.

In China, top-tier hospitals (3A-hospitals) are the highest level of hospitals according to the standard of classification. Eighty-eight percent of hospital visits by taxis are to top-tier hospitals, as reported by Didi Media Research Institute & CBNData [46]. This suggests that it is feasible to analyze the travel behavior of top-tier hospitals by taxi trajectory data. There were ten top-tier hospitals in Shenzhen by the end of 2014 according to Shenzhen Statistical Yearbook and all of them are the primary facilities covered by medical insurance (Table 1), which means that residents can choose any of these top-tier hospitals and get reimbursement for the same service at the same rate. The distribution of these ten top-tier hospitals and the population density of the study area are shown in Figure 4. The size of the yellow circle in Figure 4 denotes the number of physicians and the color of the sub-districts denotes their population density from low (light shades) to high (dark shades).

### 3.2. Data Processing

**Figure 5:**
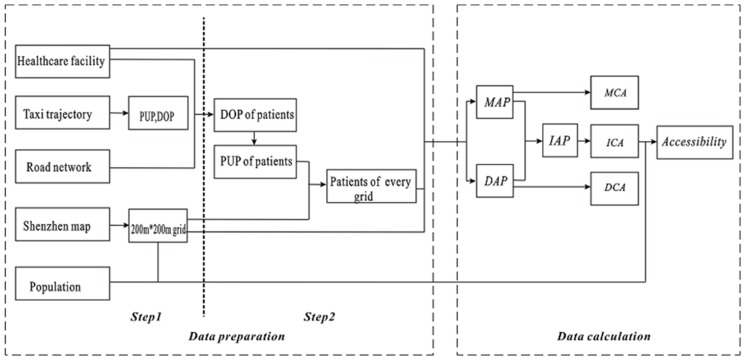
The flow of the accessibility evaluation.

As Figure 5 shows, data processing in this study was performed in two phases: data preparation and data calculation. Data preparation preceded data calculation, which is composed of two steps.

Step 1 is data pre-processing. After the basic process of transforming coordinates, removing abnormal trajectories and map matching, the taxi trajectories used in this study included 75.94 million GPS points with an average sampling interval of 40 s. Then, the trajectory data were decomposed into individual trips which began with a pick-up point (PUP) and ended with a drop-off point (DOP) [47]. Meanwhile, the minimum geographic unit available to the public (which we can obtain) is the sub-district. However, this sub-district census unit is too large for capturing the details within it. To achieve higher spatial resolution, the study area was divided into grids of 200 × 200 m and each grid cell was taken as the basic population unit. Assuming that the population is uniformly distributed within each sub-district, the population of a grid cell can be calculated and the geographic centroid of the cell is considered the population demand center. A total of 47,779 grid cells or units cover the study area.

Step 2 is mainly for extracting the patients of each grid cell. First, a rectangular buffer at the gate of each hospital was constructed for identifying and extracting patient drop-offs at the hospital’s entrance (drop-offs outside this buffer area are unlikely to be patient drop-offs from taxis; but note that one hospital seems to have several gates). The gates of the hospitals were located using Baidu map API (Application Programming Interface) tool (http://api.map.baidu.com/lbsapi/getpoint/index.html). Each buffer was set as a 100 × 20-m rectangle because we found that there are rarely other types of points of interest (POIs) within 50 m of the gates of the top-tier hospitals. Therefore, 100 m was used as the length of the rectangular buffer. Further, considering the interference of the facilities opposite a hospital, 20 m was set as the width of the rectangular buffer, which is usually smaller than the width of the road. Then, passengers with DOPs within the 100 × 20-m rectangular buffer at a hospital’s gate were selected as the patients who visited the hospital and the corresponding PUPs were identified as the patients’ home addresses. Finally, the patient number of each grid cell can be calculated by overlapping the PUPs of patients and the grid layer.

After this phase of data preparation, distances between the population units to the 10 hospitals were needed for the MAP calculation. It is important to note that, due to the nature of the raster (grid-based) spatial framework used in this study, it was normally not possible to take into account the effects of network topology and link/turn impedance between each hospital and each grid cell in the study area—since there is no obvious way in which variations in the distances among all the cells (representing network distance/time while taking topology and link/turn impedance into account) in a grid structure can be represented in ways that still allow for grid-based map algebra to operate (which operates on cell distance in the manner of “one cell, two cells or X cells apart” and thus allow for the calculation of distance based on the constant distance between any pair of cell centroids, but not on what the actual measured distance between two cells). This is a limitation all studies that examine catchment areas using a raster data structure would face and there is no satisfactory solution to the problem to date. In this case, a point-based topological network is an alternative choice to derive the distance.

Despite this limitation of the grid data structure used in our study, we implemented special procedures to utilize road network topology when estimating the MAP index. In this part of the analysis, we first constructed a topological network of the study area (which has 203,588 links and 73,515 nodes) that incorporates connectivity and directional restrictions (e.g., bidirectional or one-way roads), where road segment length was set as the link impedance. However, turn restrictions were not considered when constructing this topological network because such information was not available in the original digital road network. Using this topological road network of the study area, we mapped each of the 47,779 grid cells in the grid structure to the appropriate network node on the road network using the centroid of each grid cell. In order to derive the network-based distance matrix for estimating the MAP index, we used such mapping to transfer all cell-to-cell calculations to point-to-point network-based calculations, which was conducted using the shortest path algorithm and considering both network topology and link impedance between each hospital and each grid cell centroid. The 10 × 47,779 matrix (10 hospitals to 47,779 cells) of network travel distances was then calculated using ArcGIS. Therefore, the MAP index in this study was computed using a point-based network framework without involving any GPS trajectory data; while the DAP index was calculated using the grid-based framework using the large GPS trajectory dataset without using the topological road network because the taxi trajectories already reflect the effects of road connectivity and link impedance of the transport network in the study area. Meanwhile, the PUPs and DOPs obtained from Step 2 were used to calculate the DAP index matrix of 10 × 47,779.

Finally, in order to consider the effects of MAP and DAP equally, we set *λ*_1_ = *λ*_2_ = 0.5 in the following experiments to derive the ICAs of each hospital. These catchment areas thus reflect the link impedance of the transport network in the study area in many ways. The results and discussion of the ICA and accessibility are presented in Section 4 below.

## 4. Results and Discussion

### 4.1. Analysis of the Access Probability Threshold

Statistical results of the MAP, DAP, and IAP for each grid cell are shown in Table 2. The maximum MAP is only 0.31, which indicates that none of the hospitals has an absolute advantage in attracting patients because it is located geographically closer to them. However, the DAP ranges from zero to one, which indicates that people at the same location have similar behaviors when seeing a doctor because they may all visit a certain hospital (DAP = 1) without considering others. The minimum IAP is about half of the minimum DAP, and its maximum value of 0.66 suggests that any hospital has the probability of being visited but could not reach 100% attraction for the surrounding residents. For the convenience of comparing the results in a simpler classification, three values were selected respectively as high, middle and low levels of access probability for the MAP, DAP and IAP. That is, according to the different ranges of MAP, DAP and IAP, the high, middle and low levels of MAP were defined as 0.3, 0.2 and 0.1, the high, middle and low levels of DAP were defined as 0.9, 0.5 and 0.1, and the high, middle and low levels of IAP were defined as 0.5, 0.3 and 0.1, as Table 2 shows.

### 4.2. Analysis of the Differences among MCA, DCA, and ICA

MCA and DCA depict the catchment areas respectively from the conventional model and trajectory data. However, few studies discussed their differences and to what extent they differ from each other. In this section, we examine the differences between the MCA, DCA and ICA to explore the methodological issues concerning the measurement of accessibility.

**Figure 6:**
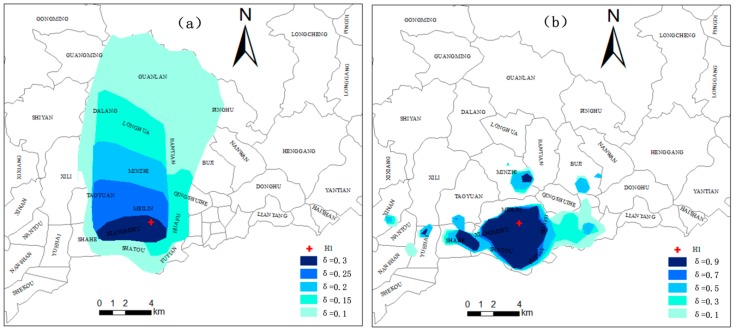
MCA and DCA of H1 under different thresholds (**a**) MCA (**b**) DCA.

Take hospital H1 as an example, the MCA at five different thresholds that represent different access probability levels (0.1, 0.15, 0.2, 0.25, 0.3) are shown in Figure 6a. Similarly, the DCA at five different thresholds (0.1, 0.3, 0.5, 0.7, 0.9) are shown in Figure 6b. The color changes from light blue to dark blue as access probability increases. At a high access probability level, the area of the MCA (H1, 0.3) is 18.34 km^2^ and the area of the DCA (H1, 0.9) is 38.15 km^2^, which means the DCA is about two times the size of the MCA. At a medium access probability level, the area of the MCA (H1, 0.2) is 83.32 km^2^ and the area of the DCA (H1, 0.5) is 60.86 km^2^, which means that the MCA is only slightly larger than the DCA. At a low access probability level, the area of the MCA (H1, 0.1) is 310.27 km^2^ and the area of DCA (H1, 0.1) is 90.61 km^2^, which indicates that the MCA is more than 3 times the size of the DCA. In addition to the large differences in size, the differences in shape between the MCA and DCA are also significant. Figure 6a shows that with the decrease in *δ*, the MCA mainly expands to the north of H1, which means that the users of this hospital are mainly distributed in areas in the north of H1 based on a largely conceptual understanding of accessibility (i.e., influenced mainly by distance). However, the DCA (Figure 6b) shows that distribution of the actual users of this hospital is extended in the east-west direction of H1, which is consistent with the distribution of the population in this region.

To further illustrate this gap, the difference in area between the MCA and DCA is represented as Δarea which can be obtained using Formula (11):

(11) $$\Delta area=area_{MCA}-area_{DCA},$$

In Formula (11), $area_{MCA}$ and $area_{DCA}$ denotes the area of the MCA and DCA respectively.

**Figure 7:**
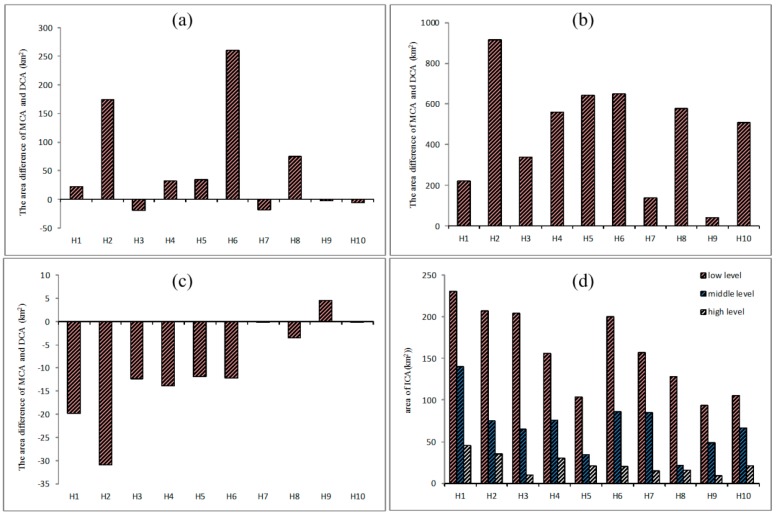
(**a**) Area differences of MCA and DCA at low level; (**b**) Area differences of MCA and DCA at middle level; (**c**) Area differences of MCA and DCA at high level; (**d**) The area of ICA at low, middle and high level.

The Δarea of these 10 top-tier hospitals at low, middle and high levels of probability thresholds are shown in Figure 7a–c. Note that at the low probability level (Figure 7a), all of the ten Δarea are greater than zero, which means all of the MCA are larger than the DCA, and it also indicates that MCA tend to overestimate the catchment areas of healthcare facilities at low levels of probability thresholds. At the middle probability levels (Figure 7b), the Δarea value of more than half of the hospitals is above the horizontal axis, which indicates the MCA is slightly larger than the DCA at middle probability levels. At the high probability levels (Figure 7c), the Δarea value of nine hospitals is much less than zero, which indicates that MCA tends to underestimate the catchment areas of healthcare facilities at high levels of probability thresholds. The overestimation or underestimation indicates that catchment areas based on conventional accessibility models do tend to deviate considerably from the catchment areas that are delineated based on real hospital visit data and thus need adjustments in practical applications.

The size of the ICA at low, middle and high levels of probability thresholds are shown in Figure 7d, which indicates that the size of the ICA varies across the 10 hospitals. This means that the size of the ICA varies by the location of a hospital. In the case of low levels of access probability, the area of H1 is the largest, followed by H2, H3 and H6, and then H4, H7, H8, H9 and H10. H1 is the most famous hospital in Shenzhen which can attract patients from far away. Therefore, its largest ICA corresponds well to the actual situation. This phenomenon also reveals that the more attractive a service facility is, the larger catchment area it has, which was proposed by Dony [20]. Similar reasons hold for H2 and H3. However, H6 is not as famous as H1, H2 and H3, but its ICA is approximately the same size as that of H3. This is mainly because H6 is the only top-tier hospital in Longgang district and it is far from other top-tier hospitals (see Figure 4). That is, people in Longgong district have few choices when seeking medical treatment. This also indicates that there is much less competition between H6 and other hospitals. For example, H4 (in Nanshan district) and H5 (in Baoan district) have similar reputations as H6 in their respective district, but H4 and H5 are close to each other and not far from Futian district which has four top-tier hospitals. Thus, the competition from the surrounding top-tier hospitals of H4 and H5 is more intense than the potential competitors of H6 which are located far away. Moreover, Longgang lies in the urban fringe and its urbanization level is lower than other districts. Therefore, a large ICA for H6, which is located in the urban fringe where medical resources are scarce, seems reasonable. This is also consistent with the findings of previous studies that healthcare facilities in the fringe of metropolitan areas or larger rural communities would frequently serve populations located well beyond the local community [8,14].

Therefore, our result shows that the size of the catchment areas varies by the location of a hospital. In addition, the attractiveness of the service facilities and the competition among them also affect the size of the service catchment areas.

**Figure 8:**
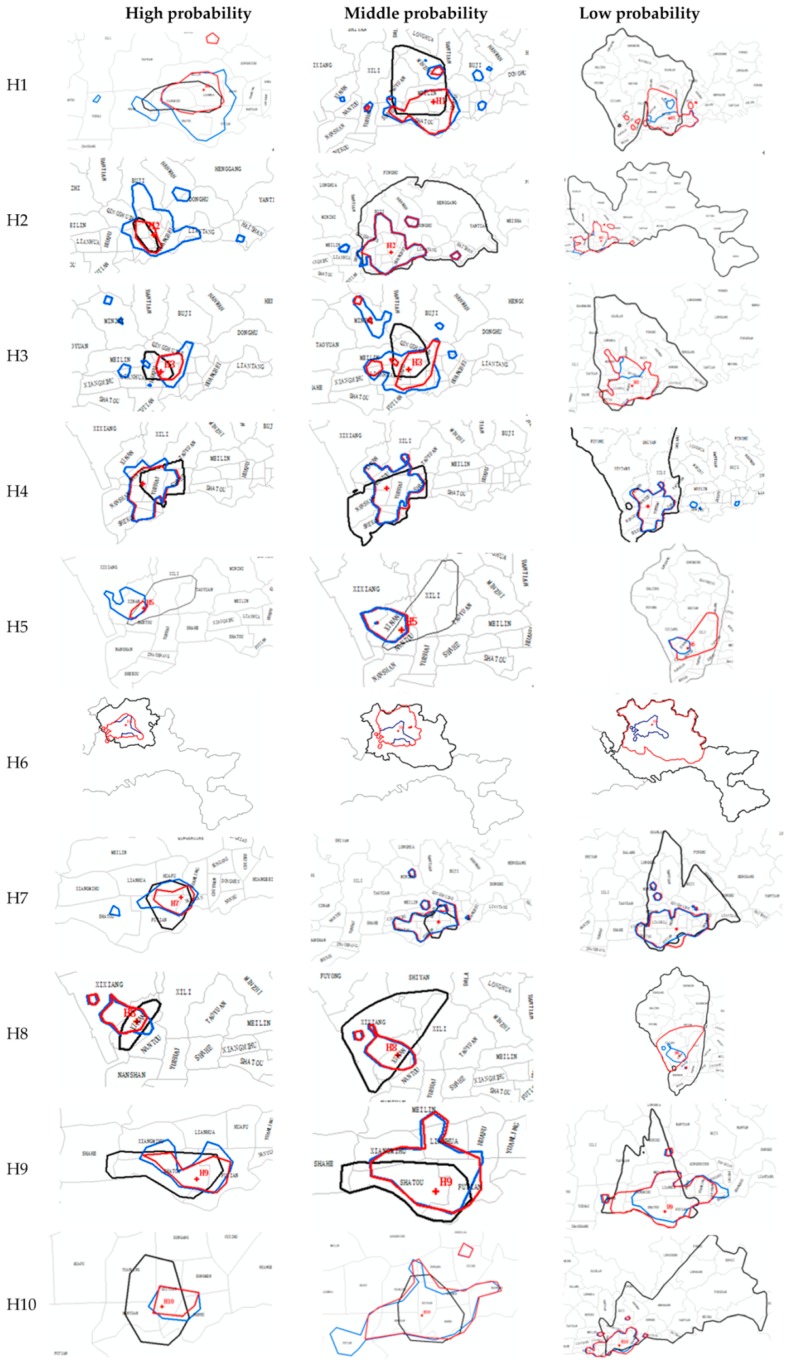
The boundary of the MCA, DCA and ICA at a high, middle and low level of probability threshold. (black line represents MCA boundary, blue line represents DCA boundary, red line represents ICA boundary).

The MCA (black line), DCA (blue line) and ICA (red line) of the 10 top-tier hospitals at three different access probability levels are shown in Figure 8. It can be seen that most of the MCA are more regular than the DCA in terms of shape. This is mainly because the MCA only considers distance but the DCA reflects complex human travel and activities. Furthermore, we find that at a high threshold level, the ICA are always at the intersection of the MCA and DCA. At a middle threshold level, the ICA are always similar to the DCA in shape. Moreover, at a low threshold level, the ICA is the result of the comprehensive adjustment of the MCA and DCA, but there are still many ICA (e.g., H2, H3, H4, H6, H7, and H10) that are very similar to the DCA in shape. This reflects two facts: (1) The ICA corrected the deviation of the MCA effectively, no matter what the access probability threshold is, the ICA can integrate the MCA and DCA in a way that obtains a shape and size that better reflect real hospital visit patterns; and (2) the DCA has a greater impact on the ICA than the MCA. In Section 4.1, the same weight (*λ*_1_ = *λ*_2_) is allocated to the MCA and DCA. In other words, the effects of the MCA and DCA on the ICA are given the same weight and considered equally in the experiment, but the ICA is still more similar to the DCA in shape, especially at a middle probability threshold level. This further illustrates the capability of the DCA in capturing more realistic catchment areas when compared to catchment areas based on conventional models of accessibility.

### 4.3. Analysis of the Characteristics of the ICA

**Figure 9:**
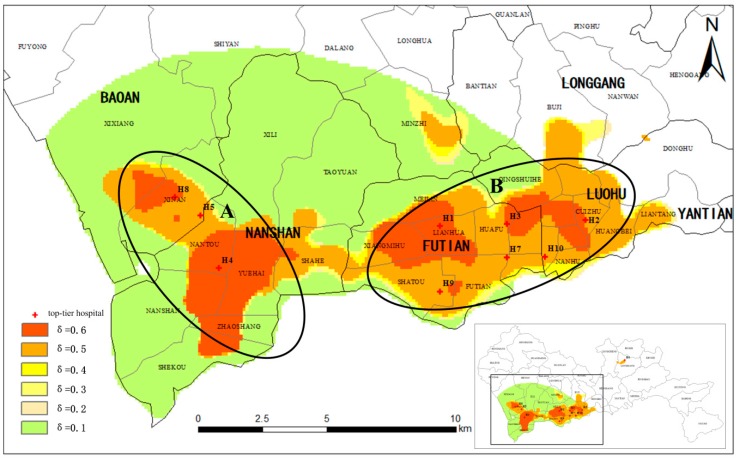
The ICA of the top-tier hospitals under different probability thresholds.

In this section, we further analyze the characteristics of the ICA. The ICA of the top-tier hospitals under six different access probability thresholds (0.1, 0.2, 0.3, 0.4, 0.5 and 0.6) are shown in Figure 9.

Three salient features stand out in Figure 9. First, when *δ* = 0.6, six hospitals (H1, H2, H3, H4, H8 and H9) have completely independent ICA. This indicates that these hospitals have great influence and reputation because they have the highest access probability for patients. In other words, these are areas where loyal users of these healthcare facilities are located. With the prominence of these hospitals and their loyal users, these areas can be viewed as the core catchment areas of these six hospitals. Second, as *δ* decreases from 0.5 to 0.2, the ICA begin to overlap and two distinct regions appear (A and B region in Figure 9). Region A includes Nantou, Yuehai, Zhaoshang, Shekou, Nanshan, Shahe, Xixiang, and Xi’nan sub-districts, and Region B includes sub-districts of Guiyuan, Nanhu, Dongmen, Sungang, Cuizhu, Dongxiao, and Huangbei sub-districts. Further, the figure shows that the size and shape of these two regions change little when *δ* varies in the range of 0.5 to 0.2. This reveals that the two catchment areas remain stable with the change in access probability. Therefore, these two regions are the stable catchment areas of the six hospitals. Third, when *δ* decreases to 0.1, the catchment areas increase so rapidly that the ICA of the hospitals all overlap together to yield a fan-shaped area, which includes the south of Baoan district, Nanshan district, Futian district, and the west of Luohu district. In this large fan-shaped area, the low access probability suggests that residents are not sure which hospital they would access. So, this region is regarded as the uncertain catchment area of the healthcare facilities. Finally, when *δ* is less than 0.1, the catchment areas extend to the whole area of Shenzhen (the blank area of Figure 9). Due to the lowest access probability and remote distance from the top-tier hospitals, this big region is regarded as the remote catchment area of the top-tier hospitals.

**Figure 10:**
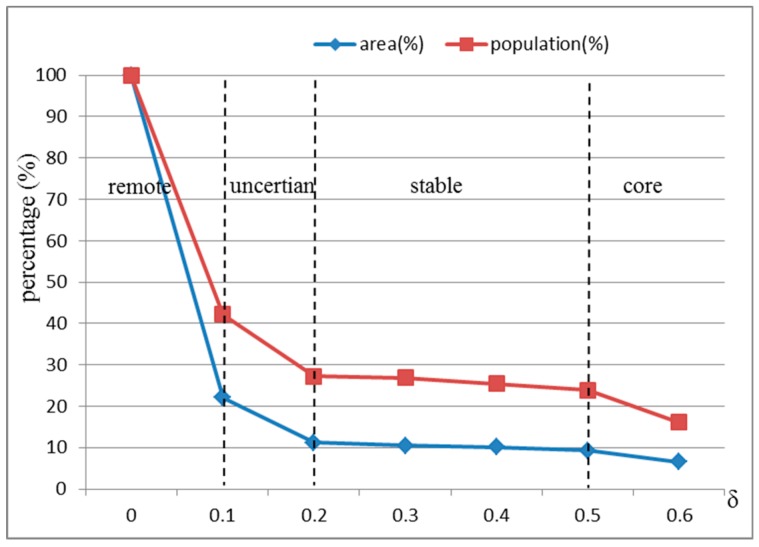
The population and area percentage of ICA under different access probabilities.

Therefore, we observe a core catchment area, a stable catchment area, an uncertain catchment area and a remote catchment area of the top-tier hospitals in the study area through three dividing points of the IAP thresholds, which are *δ* = 0.5, *δ* = 0.2 and *δ* = 0.1. These three dividing points are more evident in Figure 10, which shows the percentage of the population and area of the ICA under different access probabilities (the percentage is the proportion of the population or area of the ICA of the population or area of the entire area of Shenzhen). The red line represents the percentage of population within the ICA at six IAP thresholds, and the blue line represents the percentage of areas of the ICA at six IAP thresholds. It can be seen that the two curves both drop sharply when *δ* is less than 0.1, then, their rates of descent become slower from *δ* = 0.1 to *δ* = 0.2, and then they almost become flat curves in the range of 0.2 to 0.5. Finally, the rate of decline accelerates again when *δ* is larger than 0.5.

### 4.4. The Accessibility of the Top-Tier Hospitals

**Figure 11:**
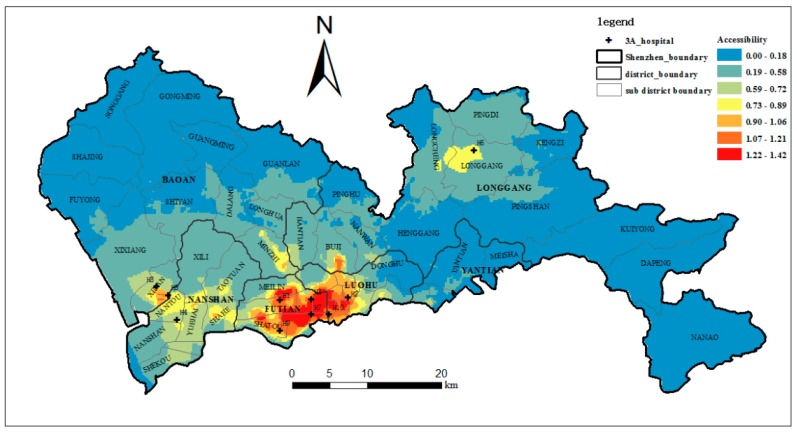
The accessibility of the top-tier hospitals (E2SFCA-ICA).

**Figure 12:**
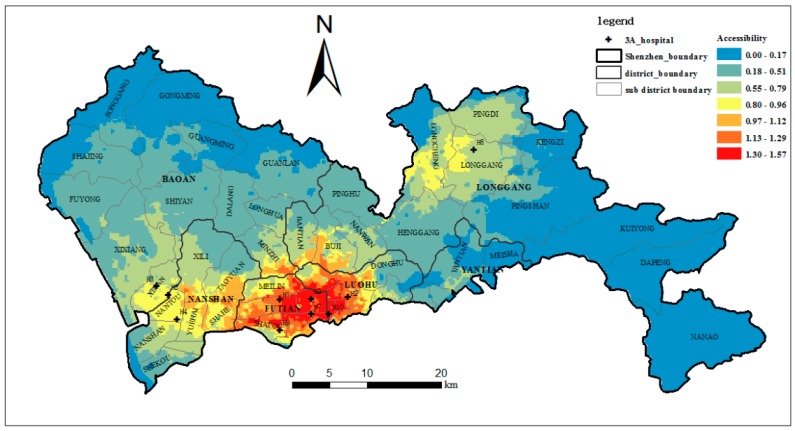
The accessibility of the top-tier hospitals (E2SFCA).

**Figure 13:**
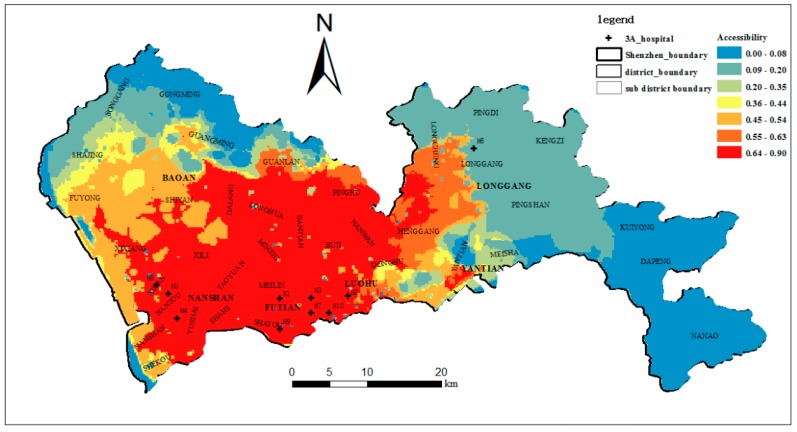
The accessibility of the top-tier hospitals (2SFCA).

In this section, to compare our method with the traditional methods, the accessibility of the ten top-tier hospitals in the study area is calculated using the E2SFCA method based on the ICA, the E2SFCA method and the 2SFCA method. According to the results of Section 4.3, four regions are formed as the IAP decreases. Therefore, the catchment area can be divided into 4 subzones of *δ* > 0.5, 0.2 ≤ *δ* < 0.5, 0.1 ≤ *δ* < 0.2 and 0 < *δ* < 0.1. In the traditional E2SFCA method, a 30-min driving zone divided into three subzones was often used as the catchment [10]. In later studies, a 60-min catchment area and four subzones were also used [48,49,50,51]. To correspond to the four subzones of ICA, a 60-min catchment area and four subzones of 0–10, 10–20, 20–30, and 30–60 min were used for the E2SFCA method, and 60-min catchment areas were used for the 2SFCA method. The Gaussian function, which has been shown to be superior to other functions, was adopted as the distance impedance function. Following McGrail [14], the distance decay weights of 1, 0.80, 0.55, and 0.15 were used for the four subzones. The accessibility based on the E2SFCA-ICA, the E2SFCA and the 2SFCA are shown in Figure 11, Figure 12 and Figure 13.

**Figure 14:**
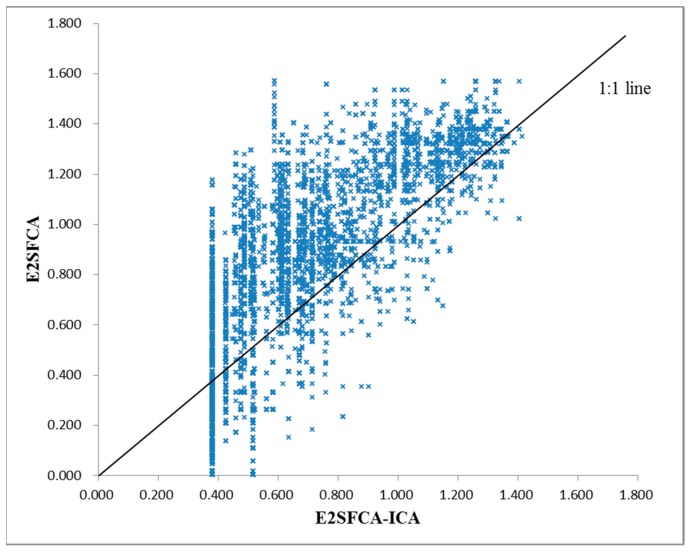
E2SFCA-ICA and E2SFCA comparison.

As shown in Figure 11 and Figure 12, and compared to using the E2SFCA method, high-accessibility areas shrink and the low-accessibility region increases when using the E2SFCA-ICA method. This indicates the travel time-based catchment areas used in the E2SFCA method tend to overestimate accessibility when compared to the ICA proposed in this study. This seems to suggest that 60-min catchment areas may lead to the overestimation of the number of accessible healthcare facilities. In traditional travel time-based catchment areas, all hospitals within a 60-min catchment area are lumped together as the supply of that catchment area. However, people actually have certain perceptions about the top-tier hospitals and the hospitals in the 60-min catchment area may not always be the final choice of the residents. As a result, the number of hospitals within the ICA is less than those found within the traditional 60-min catchment areas. The overestimation of hospital number with traditional methods thus tends to lead to the overestimation of the number of accessible healthcare facilities, which in turn results in the overestimation of the accessibility of these facilities. Accessibility scores are plotted in Figure 14 to compare the accessibility levels obtained when using the travel time-based E2SFCA method and the ICA-based E2SFCA method. The figure shows that most of the points fall above the 1:1 line, which further indicates that the E2SFCA method using the traditional catchment area tend to overestimate accessibility when compared to the ICA-based E2SFCA method.

Meanwhile, the accessibility of the top-tier hospitals is highly unequal over space. Figure 11 shows that high-accessibility areas are mainly concentrated in Luohu and Futian districts. The reason for this is that most of the top-tier hospitals are concentrated in these two districts (six out of ten hospitals), especially famous hospitals such as H1, H2, and H9. Great reputation, numerous physicians, and short distances lead to higher accessibility of this region. This also reveals how the unequal distribution of healthcare resources influences the unequal access to healthcare services in the study area [52]. Furthermore, we found substantial differences in the spatial distribution in areas with a shortage of top-tier hospitals when applying these two methods. Clearly, accessibility based on the ICA is more unequally distributed over space when compared to the spatial pattern obtained with the traditional method. This indicates that through integrating the actual visit behaviors of the residents, the uneven spatial distribution of accessibility to top-tier hospitals is aggravated. The percentages of the area and population with below-average accessibility were 62.4% and 42.2% with the traditional method, and 80.1% and 61.9% with the ICA method. This means that there are more people in a larger area with poor accessibility to the top-tier hospitals, and approximately 80% of the areas have below-average accessibility. This result is more consistent with our knowledge about the actual situation in Shenzhen, especially in sub-districts such as Shajing, Guanlan, and Longhua, whose population density is relatively high, but the accessibility to the top-tier hospitals is really low.

The unequal distribution of accessibility is more obvious when comparing the E2SFCA-ICA method with the 2SFCA method. As shown in Figure 13, the high accessibility region generated by the traditional 2SFCA method is much larger than that generated by the E2SFCA-ICA method. This is because not only real hospital visit behaviors but also distance decay are not considered in the 2SFCA method. In this case, detailed behavioral differences within the 60-min catchment area cannot be revealed and a large region of high accessibility is generated. Accordingly, the percentages of the area and population with below-average accessibility decreases to 48.9% and 24.4% when compared to the 80.1% and 61.9% obtained by the E2SFCA-ICA method. Further, comparing the sub-districts with high population density in Figure 4 with the regions with high accessibility in Figure 13, we can find that they almost completely overlap. That is to say, based on the traditional 2SFCA method, although the distribution of accessibility to top-tier hospitals is unbalanced, three-quarters of the people have above-average accessibility and the high-accessibility areas cover the high-population density regions well. However, the result by the E2SFCA-ICA method is not so optimistic. This demonstrates that the accessibility generated by the traditional 2SFCA method seems quite high throughout the city, while the accessibility that integrates real hospital visit behaviors reveals more uneven distribution. This is consistent with the results of Casas et al. [21] that “the potential access to a healthcare facility appears quite even throughout the city, the revealed access paints a very different picture.”

## 5. Conclusions

Numerous previous studies focused on determining suitable catchment areas when evaluating the accessibility of healthcare facilities. However, few studies have explored the methodological issues concerning the measurement of accessibility using model-based and data-based methods. It is worth noting that, based on the results of this study, considerable differences may exist between catchment areas obtained with traditional models of accessibility and those derived from people’s actual hospital visits. In this paper, the integrated accessibility probability (IAP) that integrates the access probability from the model-based approach and the data-based approach is presented to formulate integrated catchment areas and subzones for improving the assessment of healthcare accessibility. Some important conclusions of this paper are: (1) the proposed hybrid E2SFCA-ICA method can meaningfully divide the catchment area of a healthcare facility into four subzones (core catchment areas, stable catchment areas, uncertain catchment areas, and remote catchment areas), which are useful for understanding the distribution of healthcare visits in different regions of a study area; and (2) the traditional travel-time based catchment areas used in the E2SFCA method tend to overestimate healthcare accessibility. Furthermore, the accessibility of the top-tier hospitals in Shenzhen is more unequal over space when compared to the spatial pattern of the accessibility obtained with traditional methods.

Due to the difficulty in obtaining data about the overall utilization of public facilities, pertinent taxi trips were extracted from taxi GPS trajectories and used to represent people’s visit to the high-level hospitals in the study area. In this manner, peoples’ actual access behavior was taken into account based on the taxi trajectory data in this study, and then actual access probability was derived from the actual number of hospital visitors as a component of the integrated access probability. This is an attempt to evaluate accessibility using taxi GPS data in the absence of actual patient data. Although the patients who travel by taxi are part of the real patients of a hospital, the results reveal the effect of actual patient travel behaviors on hospital catchment areas. Previous studies found that evaluations based on different travel modes (e.g., bus, private car, biking, or walking) will lead to different accessibility results [20]. This is mainly because travel speed and travel time vary between different travel modes. So, if data of other travel modes used to visit the hospitals are available, the study can use the method to derive catchment areas using these multimodal travel data and provide a more complete picture of the service areas of the hospitals. Further, our method can be used to integrate hospital visit data based on different transport modes. In our method, the DAP index is denoted as the percentage of patients who actually visited a facility among all patients that originated from one location. It can be seen that the DAP index mainly focuses on the origin and destination of the actual patient and does not depend on a particular travel mode. Therefore, if other transport modes of actual patient data are available, the origin and destination of the patients can be extracted and then directly applied to the DAP index to enhance the accuracy and completeness of the results. This means that our approach can be adapted for evaluating the accessibility of healthcare facilities in areas with various multimodal trajectory datasets.

In addition, the ICA will have different sizes and characteristics in different applications, which is directly related to the spatial distribution of the service facilities and the specific behaviors of the people visiting these facilities. For example, with the decrease in the IAP, the ICA shows four distinct subzones at three turning points in this study. Accordingly, the ICA is divided into four subzones through these three turning points. In practical applications, the size of the ICA and the division between each subzone should be determined after analyzing the characteristics of the ICA. This is also consistent with previous views that the catchments in different areas should have different sizes [9,20,53].

Although the paper provides a novel method for integrating the model-based and data-based methods when evaluating the accessibility of healthcare facilities, several limitations should be addressed in future work. In particular, it is recognized that time is also an important factor that will influence people’s use and visit behavior with respect to healthcare services [54]. For instance, people may have different choices of healthcare providers at different times (holidays or work days, peak or non-peak periods). Therefore, more studies should be carried out using an integrated space-time framework. Furthermore, the trajectory data were only used to derive the size of the catchment areas and subzones in this study. Further studies are needed to determine the proper data-based distance decay function within each subzone. Lastly, it is also important to note that, due to the nature of the raster (grid-based) spatial framework used in this study, it was not possible to take into account the effects of network topology and link/turn impedance between each hospital and each grid cell in the study—since there is no obvious way in which variations in the distances among all the cells (representing network distance/time while taking link/turn impedance into account) in a grid structure can be represented in ways that still allows for grid-based map algebra to operate (which operates on cell distance like one cell or two cells apart, not what the distance between two cells is). This is a limitation all studies that examine catchment areas would face and there is no satisfactory solution to the problem to date. Despite this limitation, our study used 75.94 million GPS points from 12,448 taxis in the study area to derive the E2SFCA-ICA catchment areas. These catchment areas thus reflect the link and turn impedance of the transport network in the study area in many ways, since they are real taxi trajectories that had been subject to real-world network impedance.

## Figures and Tables

**Table 1:** The top-tier hospitals of Shenzhen.

Hospital Name	Abbreviation
Peking University Shenzhen Hospital	H1
Shenzhen People’s Hospital	H2
The Second People’s Hospital of Shenzhen	H3
The SIXTH people’s Hospital of Shenzhen	H4
The Eighth People’s Hospital of Shenzhen	H5
The Ninth People’s Hospital of Shenzhen	H6
Shenzhen Traditional Chinese Medicine Hospital	H7
The Second Traditional Chinese Medicine Hospital of Shenzhen	H8
Shenzhen Maternity & Child Healthcare Hospital	H9
Shenzhen Pingle Orthopedic Hospital	H10

**Table 2:** The statistical results and levels of model-based access probability (MAP), data-based access probability (DAP) and integrated access probability (IAP).

	MinValue	MaxValue	Std.	High Level	Middle Level	Low Level
MAP	0.000085	0.314345	0.038918	0.3	0.2	0.1
DAP	0	1	0.053472	0.9	0.5	0.1
IAP	0.000042	0.656681	0.036113	0.5	0.3	0.1
